# A New Method for Measuring the Rotational Angles of a Precision Spherical Joint Using Eddy Current Sensors

**DOI:** 10.3390/s20144020

**Published:** 2020-07-20

**Authors:** Penghao Hu, Linchao Zhao, Chuxin Tang, Shanlin Liu, Xueming Dang, Yi Hu

**Affiliations:** School of Instrument Science and Opto-electronic Engineering, Hefei University of Technology, Hefei 230009, China; hupenghao@hfut.edu.cn (P.H.); zhaolinchao@mail.hfut.edu.cn (L.Z.); tangchuxin@mail.hfut.edu.cn (C.T.); liushanlin0321@263.net (S.L.); dangxm@hfut.edu.cn (X.D.)

**Keywords:** spherical joint, eddy current sensor, GRNN algorithm, angle measurement

## Abstract

Precision spherical joint is a spherical motion pair that can realize rotation with three degrees of freedom. This joint is widely used in robots, parallel mechanisms, and high-end medical equipment, as well as in aerospace and other fields. However, the rotation orientation and angle cannot be determined when the joint is in passive motion. The real-time determination of the rotation orientation and angle is crucial to the improvement of the motion control accuracy of the equipment where the joint is installed in. In this study, a new measurement method that utilizes eddy current sensors is proposed to identify the special features of the joint ball and realize angle measurements indirectly. The basic idea is to manufacture the specific shape features on the ball without affecting its movement accuracy and mechanical performance. An eddy current sensor array is distributed in the ball socket. When the ball head rotates, the features on the ball opposite to the sensor, as well as the output signal of every eddy current sensor, change. The measurement model that establishes the relationship between the output signal of the eddy current sensor array and the rotation direction and angle of the ball head is constructed by learning and training an artificial neural network. A prototype is developed using the proposed scheme, and the model simulation and feasibility experiment are subsequently performed. Results show that the root mean square angular error of a single axis within a range of ±14° is approximately 20 min, which suggests the feasibility of the proposed method.

## 1. Introduction

Spherical joints support 3D rotation motions, and therefore are extensively utilized in robots, parallel mechanisms, automobiles, and medical devices due to their smooth motion, compact structure, and high load capacity. The real-time and accurate detection of the orientation and rotating angle of spherical joints is beneficial for achieving the real-time and closed-loop control of equipment and improving the control accuracy. Therefore, the methods for the real-time determination of the rotation angle of spherical joints have attracted the attention of local and international researchers.

Kok-Meng Lee et al. measured the angle of rotation of a variable-reluctance spherical motor through the visual measurement method. Concentric hemispherical shell and sphere were arranged on the outer axis of a spherical rotor, and a grid pattern similar to the globe’s longitude and latitude was sprayed on the former to encode the position information of the spherical rotor. The pseudo-random binary sequence was used to select the spacing of the grid lines to ensure no duplicates in the entire grid line sequence. The measurement resolution was dependent on the grid density. The rotation angle was finally obtained by identifying the grid coding information through visual measurement technology [1,2]. Gregory S. Chirikjian sprayed black and white stripes on the rotor surface in accordance with a certain rule in binary coding and installed an optical sensor in the stator to detect the change in the stripe color signal on the spherical surface to calculate the rotor rotation angle. The results showed that the measurement accuracy reached 1° [3,4]. Homer L. Eaton et al. designed a new style of flexible arm coordinate-measuring machine using a special spherical joint. Numerous dots or points were sculptured and scattered on the ball surface to form adjacent triangles. Each triangle, which has a unique shape and size, was correlated to a particular location in the ball surface. An optical camera in the socket was used to capture the photo that was utilized to calculate the angle [5].

The abovementioned measurement methods are based vision inspection; there are several limitations, such as low measurement accuracy, poor anti-interference ability, complex structure, and large size. To overcome these drawbacks, many scholars have adopted methods that involve magnetic effect instead of optical approaches. Compared with the latter, the former has the advantages of passivity, permeability, and invariance to environmental factors and is free of the “line of sight” requirement. W. Wang and J. Wang developed a 3-DOF spherical actuator with a four-pole spherical permanent magnet (PM) rotor, which was formed using two pairs of parallel magnetized quarter spheres. The changes in the magnetic field in space were measured by configuring multiple sensors when the rotor rotated, and the position information of the rotor was obtained using the spatial mathematical operation of the data [6].

Shaohui Foong et al. presented a direct vector field-based sensing method to measure the position/orientation of spherical joints. Twenty-four low-cost cylindrical PMs were embedded in the ball head, and two types of multi-axis magnetic sensors, namely, three-axis Hall-effect (bipolar) and two-axis giant magnetoresistance (unipolar) sensors, were fixed on the stator platform to detect the magnetic field changes caused by the ball joint rotation. The mapping relationship between the magnetic field changes and the instantaneous position of the ball joint rotor was obtained using an artificial neural network (ANN) algorithm [7,8]. Wang Wen et al. proposed a method for measuring the rotation angle of spherical joints through a spherical capacitance sensor. A central capacitor plate was embedded in the ball head, and a plurality of capacitor plates were distributed on the ball socket. When the ball output lever was in a vertical upward initial position, the capacitance values of the three capacitor plates were equal. As the spherical hinge rotated, the effective positive area detected by the three induction electrode plates changed due to the variable area measurement principle of the capacitance sensor. The capacitance values of the three measuring capacitors eventually changed. Subsequently, the relationship between the sensor capacitance value and the spherical joint space rotation angle was established to determine the rotation angle of the spherical joint in the direction with two degrees of freedom [9,10,11,12].

**Figure 1:**
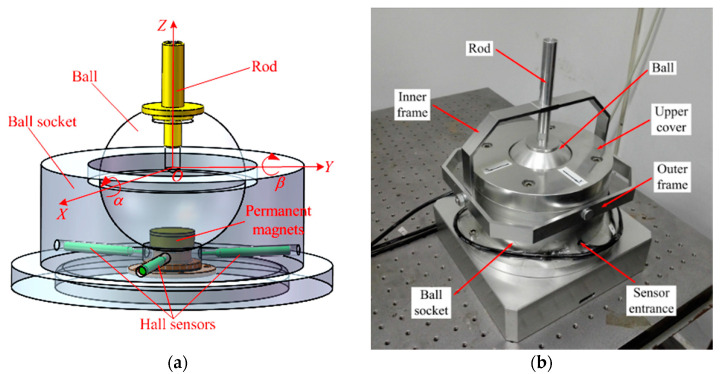
Prototype of the intelligent spherical joint; (**a**) structure diagram; (**b**) prototype.

A magnetic effect method to measure the rotation angle of a precision ball joint has also been developed in our team. A cylindrical PM was embedded on the ball, and three Hall sensors were unevenly arranged in the ball socket (Figure 1a). When the ball arbitrarily rotated in the ball socket, the magnetic field rotated synchronously in space, which caused the magnetic induction intensity at the sensor position to change. The Hall sensor array captured the variation. The angles of rotation about the x and y axes, which are respectively denoted as *α* and *β*, were calculated to determine the motion direction and rotation angle of the ball club. This kind of spherical joint is named “intelligent ball joint” [13,14]. 

We also established the measurement model that describes the relationship between the rotation angles (*α* and *β*) and the output of the Hall sensor in two ways. First, an analytical model was constructed on the basis of the equivalent magnetic charge model. The comparative experiment results were in the range of ±20°, the single axis angle measurement accuracy was 12′, and the resolution was 22″ [15,16]. Second, the measurement model was established using an ANN algorithm. The experimental results showed that the measurement accuracy of the prototype after using the ANN algorithm greatly improved; the range was within ±20°, the single axis measurement accuracy was 4’, and the resolution was 15” [17,18].

To further improve the measurement accuracy and resolution, we performed a systematic analysis of the error factors that affect the measurement accuracy [19]. The results revealed that the clearance of the ball joint is a crucial error factor. This clearance not only affected the working accuracy, but also caused the ball center to drift, which induced additional changes in the magnetic induction strength value at the location of the Hall sensor and resulted in angle measurement errors. Therefore, we embedded three eddy current sensors in the ball to detect the clearance in real time.

The eddy current sensor is widely used in the metal, nuclear, and aircraft industries; it can inspect electrically conductive materials at very high speeds that does not require any contact between the test piece and the sensors [20]. It can be used to measure displacement, size, shape, and runout [21]. It also can be used to detect angle [22,23] and thickness [24]. It has high precision, sensitive response and strong anti-interference capability [25].

The measured clearance data were used not only to compensate for the motion control error of the equipment at the location of the ball joint, but also can be used to correct the angle measurement error to improve the overall intelligent level of the spherical joint.

The original intention of using an eddy current sensor here is to measure the ball hinge clearance in real time. However, this action provides a new idea regarding the intentional manufacturing of a small groove, plane, or hole in the ball head as a feature. When the ball head rotates, the eddy current sensor can be used to sense its change, and the artificial neural network can be utilized to build the relationship between the rotation angle and the output of the sensors to identify the rotation direction and angle of the ball head. If this scheme is feasible, the PM and Hall sensor can be removed. Moreover, the geometric feature distributed on the ball head can also store grease, reduce the wear of the ball head, and extend the service life.

## 2. Measurement Principle Based on the Eddy Current Sensor

An eddy current sensor is typically used to obtain accurate size and distance measurements on the basis of the variation in the output voltage of the sensor caused by the back electromotive force (EMF) produced by the eddy current effect. The magnitude of the back EMF is directly related to the characteristics of the eddy current field produced by metals. Therefore, the material should be calibrated before using the eddy current sensor to measure the distance because the different compositions of the metal, as well as the different shapes, thickness, and distances of the measured body, will change the eddy current field. In other words, the eddy current sensor not only measures the distance accurately, but also produces different continuous output signals when it scans the contour of different shapes and depths. Such features suggest the feasibility of the proposed measurement method. 

**Figure 2:**
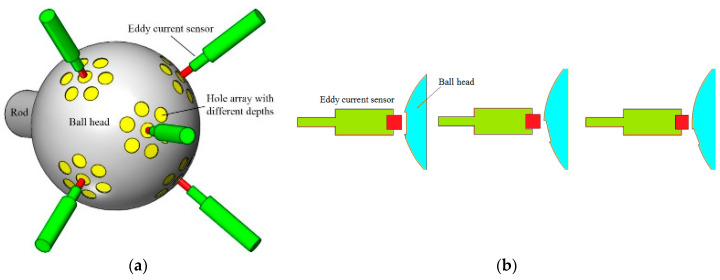
Measuring principle. (**a**) measuring principle; (**b**) working states of sensor.

The structure of the spherical joint with the new measurement principle is shown in Figure 2a. Holes with different diameters and depths are distributed on the surface of the lower hemisphere of the ball head, and several eddy current sensors are arranged in the ball socket. When the ball head rotates, each eddy current sensor faces different areas of the ball head (Figure 2b). If the sensor exactly points to a certain hole, the output signal is the hole depth. If the sensor points to a part of the hole section, the output signal of the sensor will be related to the cross-section overlap area and contour feature variation. If the sensor directly points to the ball head surface without encountering a hole, the sensor will detect the clearance of the ball joint. These conditions mean that the combination of the output results of all eddy current sensors is related to the rotation direction and angle of the ball head. On this basis, artificial neural network can be used to complete the algorithm training and learning on the calibration device to construct the measurement model.

## 3. Design of the Features in the Ball Head

**Figure 3:**
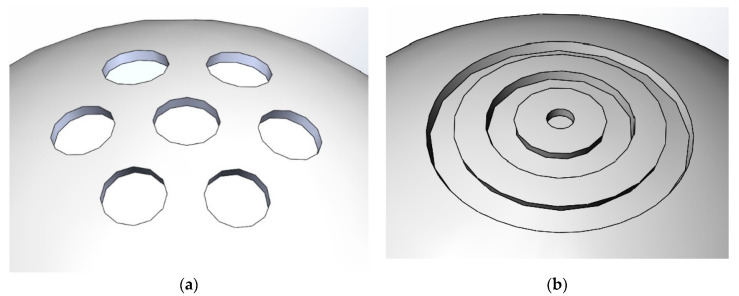
Selection of the features for the ball head; (**a**) hole shape; (**b**) groove shape.

To improve the accuracy and resolution of the proposed measurement approach, the best spherical feature for the ball head must be designed and selected. The selection is based on the following requirements. First, the feature must facilitate the eddy current sensor in obtaining information and producing continuous signal output. Second, the features on the different points at the ball head should be different from each other to effectively aid the artificial neural network in establishing the model and improve the measurement accuracy. Figure 3 shows the two proposed structures. The hole group, which is easy to manufacture and exerts minimal influence on the original structure and mechanical properties of the ball head, is selected, and relevant analysis and calculation are subsequently performed. 

### 3.1. Characteristics of the Eddy Current Sensor

**Figure 4:**
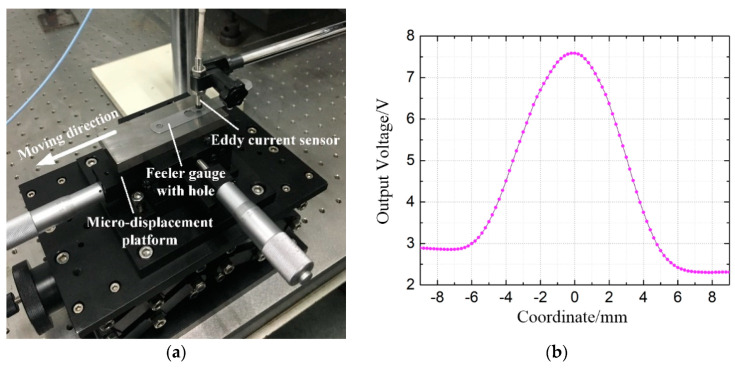
(**a**) Domestic eddy current sensor and (**b**) curve of the data change.

Before determining the depth and spacing of the holes in the hole group, the characteristics of the eddy current sensor output when scanning over a plane with holes should be investigated to determine the structural parameters of the hole group and form a good matching effect. Figure 4 displays the tested domestic eddy current sensor (type: cwy-do-tr-810303-01; probe diameter = 3 mm; output = 0–10 V; resolution = 0.05 μm). A feeler gauge (thickness = 0.2 mm) with a ∅6 mm hole is fixed on the micromotion stage. When the center line of the probe points to the hole center, the coordinate value is set to 0 and the feeler gauge is moved left and right at equal intervals of 0.2 mm on the micromotion stage. The output of the eddy current sensor is observed and recorded. The curve in Figure 4b shows that the eddy current sensor is effective and feasible in identifying the shape characteristics of the cylindrical hole. Moreover, the output signal of the eddy current shows a clear and continuous variation within the range of approximately 12 mm diameter around the hole, which greatly helps in determining the hole spacing in the hole group, as well as in establishing the artificial neural network model.

### 3.2. Determination of the Hole Group Size Parameters and Simulation Analysis

**Figure 5:**
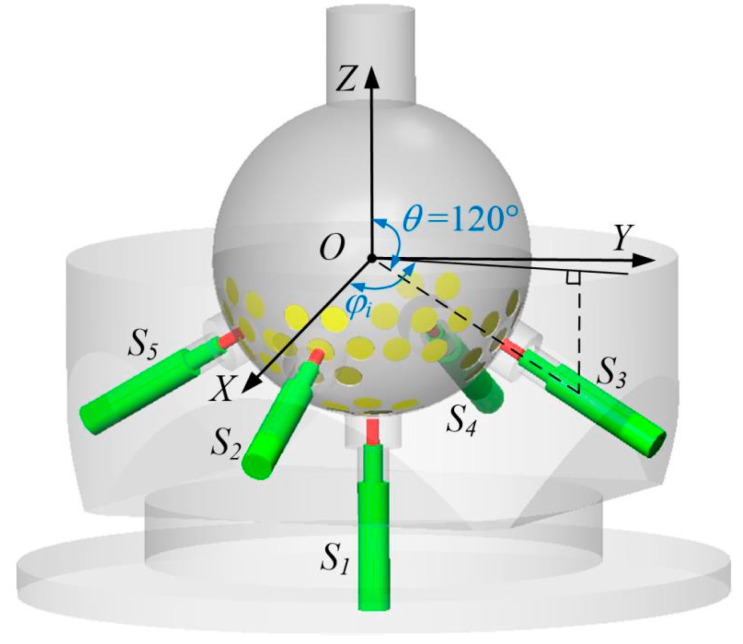
Arrangement of the hole array.

Using the data obtained in the eddy current sensor characteristic test and the experience gained from previous research on a PM–Hall sensor prototype, the preliminary design scheme of the hole group is constructed (Figure 5). A center hole is surrounded by six holes with diameters of 9 mm, and the diameter of the ball head is 100 mm. Consistent with a measurement range of ±20°, the angle between the center lines of the outer and center holes is 20°, so the arc length *l* is approximately 34 mm. The measurement range of the eddy current sensor is 0.5 mm; thus, the average depth of the hole is set to 0.4 mm. This size choice can ensure that the output signal of the sensor is only determined by the nearest hole and no other hole influence is superimposed. 

**Figure 6:**
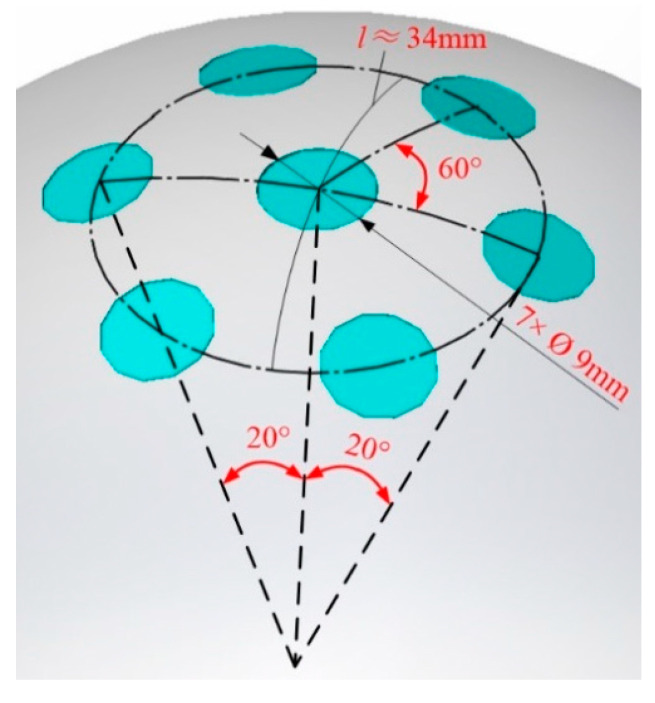
Arrangement of sensors and hole groups.

After determining the structure and size of one hole group, the number of hole groups and sensors needed in the ball joint should be calculated. Increasing the number of sensors and hole groups increases the number of the neural network inputs, and thus improves the measurement accuracy and resolution. However, the presence of excessive hole groups on the surface of the ball head will have a certain negative effect on its mechanical properties and strength. In addition, increasing the number of sensors will raise the difficulty in designing and manufacturing the ball socket. Five eddy current sensors and five hole groups are preliminarily selected. As shown in Figure 6, the center holes of the five hole groups are facing the five eddy current sensors; each sensor points to the center of the ball. The first sensor is placed on the z axis, at the lowest point of the ball socket, and the other four sensors are evenly distributed in the ball socket with a 30° inclination with respect to the *XOY* plane.

**Figure 7:**
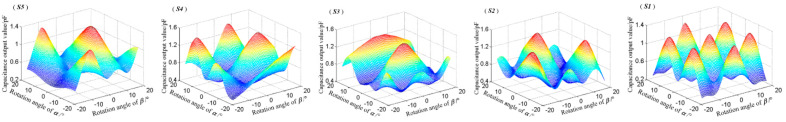
Capacitance output value distribution chart of Sensors 1–5.

On the basis of the structural parameters of the ball head, the capacitance electrostatic field module in the ANSYS Maxwell simulation software is used to simulate the eddy current sensor because the characteristics of this sensor are similar to those of the variable area capacitance sensor. *α* and *β* are assigned with values every 2° during the simulation at a range of ±20°. The capacitance outputs are recorded after the simulation (Figure 7). The capacitance distribution curve of the outputs of Sensor 1 shows seven similar peaks, and the peak distribution is consistent with the distribution of the array holes. The amplitudes of the signals of Sensors 2–5 exhibit large variations, and the characteristic trend of each sensor is distinct, which is useful for establishing the measurement model using the artificial neural network. Conversely, the presence of multiple peaks in the output is not conducive to the modeling process because it causes the training and learning procedures to converge to the incorrect range and therefore induces error. Therefore, the structure should be simplified and the number of sensors should be reduced.

**Figure 8:**
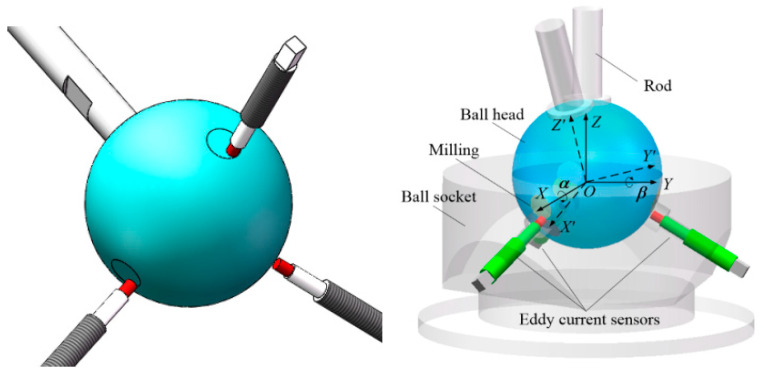
Eccentric distribution model of a single hole.

**Figure 9:**
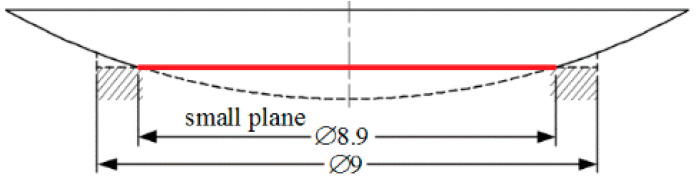
Small plane on the ball head.

Compared with the multi-hole array model, the output of the single hole design is simple and unimodal. In addition, the availability of data is greatly improved, which is beneficial for the neural network model. The same simulation analysis is then used to analyze the scheme that involves a single hole on the ball head, and the results show that such scheme is feasible. Adopting the single-hole design scheme further reduces the overall sizes of the ball head and prototype. The structure of the single-hole prototype, whose diameter is reduced to 50 mm, is shown in Figure 8. Three holes with diameters of 9 mm are drilled in the ball head. However, because the hole depth is set in accordance to the average size (0.4 mm), the hole is relatively shallow, and the shape characteristics of the ball head no longer resemble a hole and appear as three small planes with diameters of approximately 8.9 mm (Figure 9). The matched three eddy current sensors are installed in an eccentric way relative to the hole center, that is, when the center of one sensor points to the center of the small circular plane, the other two sensors are offset. This arrangement, which is based on the measurement principle of the Vernier caliper, improves the measurement accuracy.

## 4. Hardware and Software Algorithms

### 4.1. Eddy Current-Based Prototype

**Figure 10:**
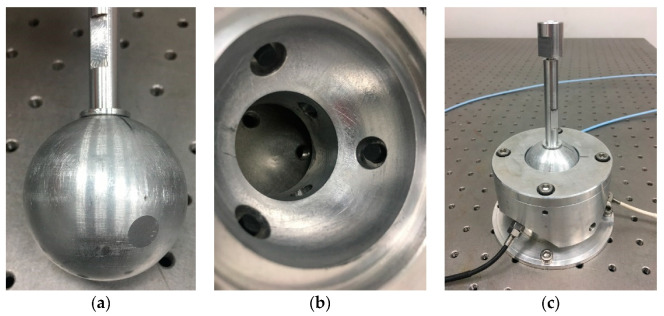
The new prototype with eddy current sensors; (**a**) ball; (**b**) ball housing; (**c**) new prototype.

The ball joint prototype based on eddy current sensors was manufactured and assembled on the basis of the analysis and measurement feasibility simulation results. As shown in Figure 10a, three small planes with diameters of 50 mm were milled on the ball head and uniformly distributed along the circumference (*θ* = 110°, see Figure 6 for the definition of *θ*). Three holes were allocated for the installation of the eddy current sensor in the ball socket (Figure 10b). These holes were evenly distributed along the circumference (*θ* = 120°), and the center line of the hole points to the ball center. The part near the inner surface of the ball socket was machined with a countersunk hole to ensure that the probe was not affected by the socket metal. The prototype is shown in Figure 10c.

### 4.2. Generalized Regression Neural Network (GRNN) Algorithm

In the previous study on intelligent spherical joints based on magnetic effect, we applied several forms of artificial neural network to build the measurement model and encountered various drawbacks. For instance, the common backpropagation artificial neural network easily falls into the local extremum when solving complex nonlinear functions, and the convergence speed during training was slow. In addition, the radial basis function artificial neural network requires a high repetition accuracy of the data samples, but often generates error on the long-distance sample points. In the present study, GRNN was selected to establish the measurement model. The final convergence result of GRNN was the optimal regression surface with the largest sample size, and the establishment of the learning samples led to the determination of the connection weight among the neurons. Moreover, the GRNN algorithm is highly suitable for small samples.

**Figure 11:**
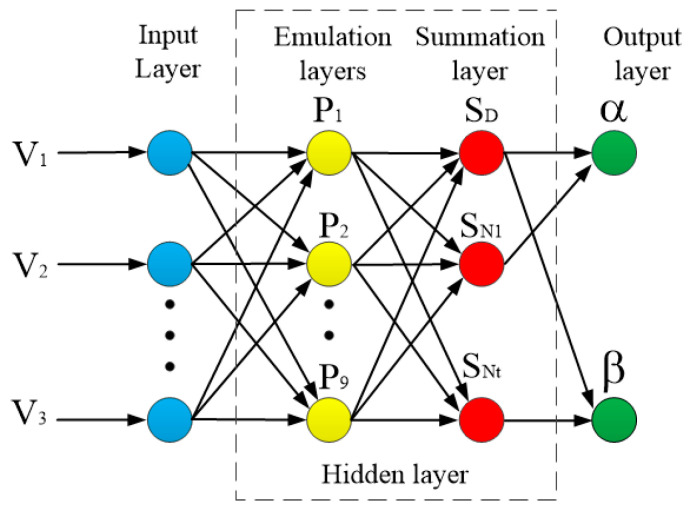
Generalized Regression Neural Network (GRNN) artificial neural network structure.

The structure of the GRNN artificial neural network is illustrated in Figure 11. The feedforward network structure was composed of the input, emulation, summation, and output layers. The number of neurons in the input layer was the same as the dimension of the input vector of the training sample. Three eddy current sensors were used (i.e., m = 3). The input layer directly passes the input variables to the pattern layer. The number of neurons in the model layer was the same as the number of training samples. The transfer function of the neurons in the emulation layer is expressed as

(1) $$R_{i}=\exp[-\frac{{\left\|V-V_{i}\right\|}^{2}}{2\sigma_{i}^{2}}]\;\;i=1,2,\cdots ,n,$$

where *V* is the output voltage of the eddy current sensor, *V_i_* is the corresponding sample of neuron *i*, and σσσσ is the smoothness factor. Two types of neurons are used in the summation layer. The first kind of formula is the arithmetic summation.

(2) $$S_{D}=\sum_{i=1}^{n}\exp[-\frac{{\left(V-V_{i}\right)}^{T}(V-V_{i})}{2\sigma^{2}}]$$

Another kind of formula is the weighted summation, which involves the output *Yi* and angles *α* and *β*.

(3) $$S_{N}=\sum_{i=1}^{n}Y_{i}\exp[-\frac{{\left(V-V_{i}\right)}^{T}(V-V_{i})}{2\sigma^{2}}]$$

The output layer *S* is the division of the weighted summation value *S_N_* and arithmetic summation value *S_D_*.

(4) $$y_{j}=\frac{S_{Nj}}{S_{D}}\;\;j=1,\;2,\;\cdots ,\;k$$

The summation and output layers complete the weighting calculation, and these layers can be collectively called the weighting layer. GRNN adopts the same smoothing factor for all kernel functions of the hidden layer units. The training of the network is a 1D optimization step, which is convenient, fast and easy to implement in the hardware.

## 5. Experiment and Error Analysis

**Figure 12:**
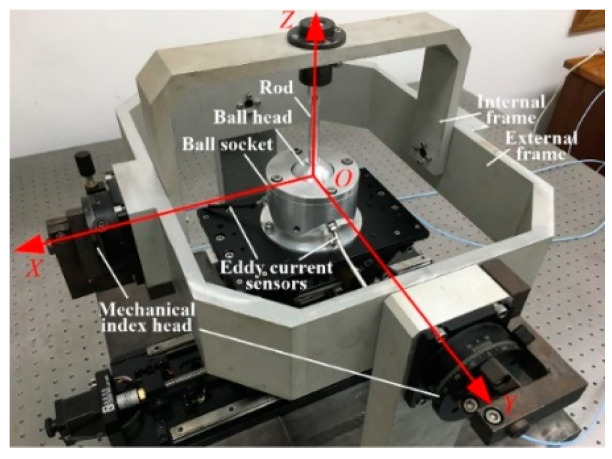
Mechanical biaxial angle platform.

The experiment consisted of two parts that were completed on the biaxial angle platform that we developed. The structure of this platform was similar to that of a gyroscope, which is composed of inner and outer frames (Figure 12). The standard angle value was given by two mechanical dividing heads with an accuracy of 3’. The first experiment involved the training and learning of the artificial neural network. During both processes, the double axis drove the ball joint to rotate step by step at a certain interval and provided the standard angle values (*α* and *β*) for the prototype. The output signals of the three eddy current sensors were recorded and formed into a number array with corresponding *α* and *β* values, to be used in the training and learning of the ANN to construct the measurement model. After the measurement model was obtained, the accuracy test of the prototype was conducted on a calibration device. The experimental process was similar to the training and learning of the prototype, but the measured value, which is the inverse solution of the measurement model, was compared with the given standard angle of the calibration device to determine the measurement error.

### 5.1. GRNN Training Test

**Figure 13:**
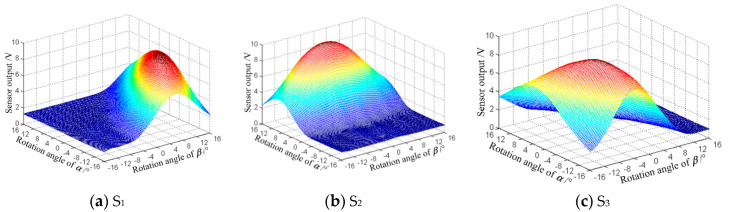
Measured voltage value of the eddy current sensors.

To improve the calculation accuracy of the measurement model, the new ball joint prototype was fixed on the 3D micromotion stage before the training and learning experiments. The prototype was fixed on the biaxial platform in advance so the ball joint center and the intersection of the two axes of the platform could be adjusted to coincide with the 3D micromotion stage. In the training experiment, the inner and outer frames were driven manually at an interval of 2° to provide a standard spherical motion for the ball joint in any orientation. The output voltages of the three eddy current sensors and the corresponding orientation angle values (*α* and *β*) were recorded synchronously to obtain the dataset needed to train and establish the measurement model. Finally, a 17 × 17 dataset was collected through the training experiment. The output of the three sensors is presented in Figure 13.

Figure 13 shows that the output signal of each eddy current sensor was unimodal, and the output laws of the three sensors were significantly different, which was consistent with the conclusion of the simulation analysis and conducive to the realization of artificial neural network modeling. After the data collection, the training and fitting program based on GRNN was inputted into the MATLAB script module to execute the training and calculation and eventually construct the measurement model.

### 5.2. Accuracy Evaluation Experiment

Given that the measurement software of the prototype was developed using LabVIEW, and the training, learning, and modeling were completed in MATLAB, the measurement model in the MATLAB script needed to be connected to the measurement software. In the error acquisition experiment, the biaxial platform rotated at a certain step and provided the standard angle value, and the measurement model calculated the results using the outputs of the three sensors. The difference between the calculated and standard angles provided by the biaxial platform is regarded as the measurement error of the prototype. Given the large amount of error data obtained in the experiment, only the partial angle error values are presented (Table 1).

**Figure 14:**
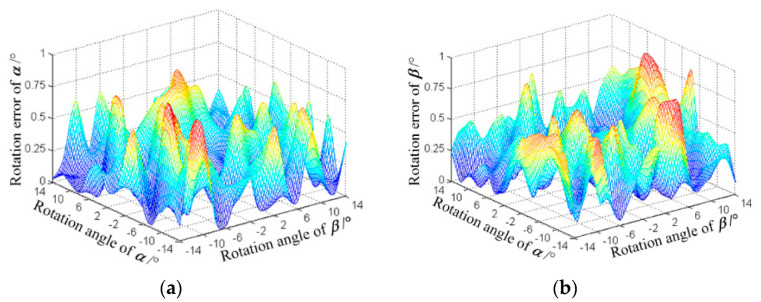
Measurement error distribution of *α* and *β*; (**a**) distribution of *α*; (**b**) distribution of *β*.

The result reveals that the actual working range was ±14°, which was less than the designed range (±20°); the measurement error deviated far from ±14°. The mean square deviation of *α* and *β* within the actual working range were 19′36″ and 23′18″, respectively. When *α* and *β* were negative, the measurement error was large, but the overall error distribution was relatively uniform. The measurement error distribution is displayed in Figure 14.

### 5.3. Analysis of the Main Error Sources

The measurement accuracy of the proposed method was relatively low, but its feasibility was confirmed by the experiments. In this section, the main error sources are analyzed to improve the measurement accuracy. These sources of error include the manufacturing and assembly of the ball joint and biaxial platform, stability of the eddy current sensor output, and calculation errors of the artificial neural network model.

(1) Ball joint clearance

The clearance in the ball joint resulted in a slight variation in the output of the eddy current sensors when the ball head rotated repeatedly at a same point and therefore produced a repeatability error. This error negatively affected the calculation accuracy of the GRNN model. In addition, the mechanical manufacturing accuracy of the developed double-axis angle platform was limited, and the low mechanical indexing accuracy restricted the measurement accuracy of the prototype. In the following research, we will reduce the clearance in the new prototype, and redevelop a two-dimensional rotary table with higher accuracy angle grating.

(2) Output drift of the eddy current sensor

The eddy current sensor had several disadvantages, such as temperature drift and linearity difference, which affected the measurement accuracy of the prototype during model training, learning, and testing. The experimental results showed that the current domestic eddy current sensor required one hour to stabilize after it was turned on. Therefore, an eddy current sensor with excellent anti-drift characteristics should be selected to improve the measurement accuracy.

(3) Error resolution for the GRNN algorithm 

Resolving the error in the algorithm of the artificial neural network is an important influencing factor of the final measurement accuracy. Therefore, the influence of the number of inputs, hidden layers, thresholds, or other functions in the algorithm on the convergence speed and accuracy should be studied to determine interconnection between the weight (or bias) of the neural element in the algorithm and the angle measurement accuracy. Moreover, the errors involved in intelligent ball joints should be resolved in real time, and the new characteristic of the calculation error of the algorithm should be identified to correct or compensate for the calculation error. 

## 6. Conclusions

The measurement method proposed in this study provides insights into an innovative approach for spherical angle measurement by manufacturing holes on the ball head. The geometric features of these can be detected using eddy current sensors. When the ball head rotates, the output signal of the eddy current sensor array changes. The measurement model is established by learning and training an artificial neural network, and information redundancy is also realized to improve measurement accuracy, and a prototype is subsequently developed. Results verify the feasibility of the proposed scheme. Given that the current measurement accuracy of the method is relatively low, this approach cannot be directly used in precision engineering. However, the proposed method transforms the understanding about angle measurement in engineering, especially regarding the principles of traditional angle sensors, which involve equally dividing the space and marking the disk, and realizing measurements using an analytical measurement model. The proposed measurement model can be established using an artificial intelligence algorithm and used in measuring the displacements and angles of objects with certain geometric or physical features on the surface (e.g., holes, slot, or magnetic field) as long as the sensor can identify these features. Furthermore, because the proposed method uses the features of the measured object to realize measurement, installing a grating ruler or grating disk on the equipment is not necessary. Future works should investigate the potential improvements for the presented measurement technique.

## Figures and Tables

**Table 1:** Measurement error distribution chart of *α* and *β* (−8° < *α* < 8°, −4° < *β* < 8°).

Δ*α*/°									Δ*β*/°								
	*β*/°	−4	−2	0	2	4	6	8		*β/*°	−4	−2	0	2	4	6	8
*α/*°									*α/*°								
−8		−46″	−8′51″	−23′57″	−51″	2′8″	9′7″	16′7″	−8		35′15″	48′54″	−29′48″	−1′22″	−8′0″	−1′2″	−21′46″
−6		−16′12″	−18′55″	−42′49″	−32′57″	−27′55″	−5′46″	17′59″	−6		28′52″	31′39″	−39′18″	−4′32″	−2′0″	−16′26″	−33′58″
−4		−11′4″	−47″	−21′28″	−21′10″	−23′45″	−14′55″	−15′14″	−4		34′39″	22′26″	−7′22″	−3′37″	−22′1″	−35′2″	−40′58″
−2		−14′14″	−8′6″	−9′58″	−4′57″	4″	−20′59″	−37′58″	−2		29′0″	20′57″	−3′35″	−2′28″	−22′9″	−29′34″	−36′0″
0		−3′36″	7′5″	−2′25″	−21″	1′28″	−4′44″	−28′29″	0		29′50″	22′47″	−1′10″	−6′6″	−22′15″	−22′22″	−20′56″
2		2′18″	20′04″	6′46″	4′21″	6′25″	5′22″	48″	2		22′0″	25′17″	2′18″	−10′34″	−22′27″	−18′26″	−11′36″
4		6′50″	14′35″	10′10″	4′8″	10′27″	6′25″	6′10″	4		14′10″	22′11″	−56″	−9′45″	−20′0″	−8′39″	−10′6″
6		22′55″	17′16″	24′30″	23′31″	14′59″	19′22″	16′13″	6		6′12″	13′2″	−1′45″	−2′30″	−14′55″	6′18″	−1′47″
8		24′31″	36′34″	41′31″	38′45″	24′49″	24′23″	12′48″	8		−15′29″	2′22″	−4′51″	3′52″	1′0″	3′40″	1′58″
